# Uif, a Large Transmembrane Protein with EGF-Like Repeats, Can Antagonize Notch Signaling in *Drosophila*

**DOI:** 10.1371/journal.pone.0036362

**Published:** 2012-04-30

**Authors:** Gengqiang Xie, Hongtao Zhang, Guiping Du, Qinglei Huang, Xuehong Liang, Jun Ma, Renjie Jiao

**Affiliations:** 1 State Key Laboratory of Brain and Cognitive Science, Institute of Biophysics, the Chinese Academy of Sciences, Beijing, China; 2 Graduate School of the Chinese Academy of Sciences, Beijing, China; 3 Division of Biomedical Informatics, Cincinnati Children's Research Foundation, Cincinnati, Ohio, United States of America; 4 Division of Developmental Biology, Cincinnati Children's Research Foundation, Cincinnati, Ohio, United States of America; National Institutes of Health (NIH), United States of America

## Abstract

**Background:**

Notch signaling is a highly conserved pathway in multi-cellular organisms ranging from flies to humans. It controls a variety of developmental processes by stimulating the expression of its target genes in a highly specific manner both spatially and temporally. The diversity, specificity and sensitivity of the Notch signaling output are regulated at distinct levels, particularly at the level of ligand-receptor interactions.

**Methodology/Principal Findings:**

Here, we report that the *Drosophila* gene *uninflatable* (*uif*), which encodes a large transmembrane protein with eighteen EGF-like repeats in its extracellular domain, can antagonize the canonical Notch signaling pathway. Overexpression of Uif or ectopic expression of a neomorphic form of Uif, Uif*, causes Notch signaling defects in both the wing and the sensory organ precursors. Further experiments suggest that ectopic expression of Uif* inhibits Notch signaling *in cis* and acts at a step that is dependent on the extracellular domain of Notch. Our results suggest that Uif can alter the accessibility of the Notch extracellular domain to its ligands during Notch activation.

**Conclusions/Significance:**

Our study shows that Uif can modulate Notch activity, illustrating the importance of a delicate regulation of this signaling pathway for normal patterning.

## Introduction

Notch signaling is an evolutionarily conserved signaling pathway that regulates a variety of different developmental processes, including adult homeostasis and stem cell development [1], [2], [3], [4]. In *Drosophila*, both the Notch receptor and its canonical ligands, Delta (Dl) and Serrate (Ser), are transmembrane proteins with large extracellular domains consisting primarily of EGF-like repeats. The canonical Notch pathway is activated by an interaction between the Notch receptor on one cell with its ligand on the neighboring cell. Such an interaction induces two consecutive proteolytic processes that result in the release of the Notch intracellular domain, which is then translocated to the nucleus and activates transcription of its target genes by interacting with the DNA-binding protein Suppressor of Hairless (Su(H)) and the coactivator Mastermind.

Several Notch receptors and a large number of Notch ligands and co-ligands have been identified in mammals and *C. elegans* [5], [6]. In *Drosophila*, a single Notch receptor and two canonical ligands, Dl and Ser, are well characterized. Recently, an EGF-repeat-containing protein, Weary (Wry), was identified as a new Notch ligand important for the maintenance of normal heart function in the adult fly [7]. The complexity of the biological processes controlled by the Notch signaling pathway requires precise regulation of its activity, particularly at the level of ligand-receptor interactions. For example, the secreted glycoprotein Scabrous (Sca) has been shown to positively modulate the Notch activity in regulating proneural development in *Drosophila* eyes [8], [9]. In addition, Crumbs (Crb), an EGF-like repeat-containing large transmembrane protein well characterized for its role in epithelial organization [10], was recently shown to act as a negative regulator of Notch signaling in the *Drosophila* wing [11]. A significant part of the complexity and specificity of Notch signaling is derived from the inhibitory action of Notch antagonists.

In this report, we describe the role of a recently identified gene, *uninflatable* (*uif*), in antagonizing Notch signaling activities when overexpressed. *uif* was initially characterized for its role in tracheal development in *Drosophila* [12]. It encodes a transmembrane protein with a large extracellular domain consisting of eighteen EGF-like repeats, a feature common to the Notch receptor and its ligands. Here, we show that Uif can antagonize the canonical Notch signaling pathway, acting at a step that is dependent on the extracellular domain of Notch. Our results suggest a model where Uif antagonizes Notch activity in a neomorphic manner by influencing the accessibility of its extracellular domain available for interacting with its ligands on neighboring cells during Notch activation.

## Results

### Ectopic expression of an altered form of Uif causes phenotypes characteristic of Notch signaling defects

**Figure 1:**
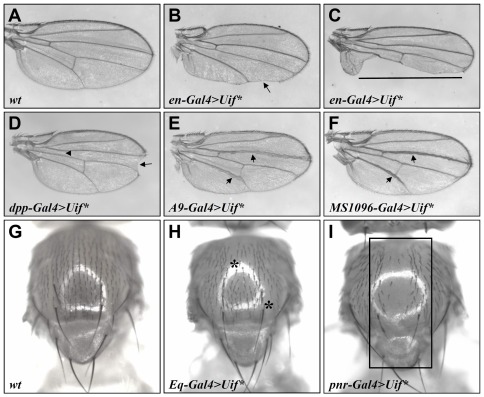
Ectopic expression of Uif* causes phenotypes that are characteristic of Notch signaling defects. (A) A wt adult wing. (B and C) Targeted Uif* expression under the control of *en-Gal4* causes loss of wing margin structures in the posterior wing compartment. These two panels show different expressivity, ranging from a partial loss of wing margin (arrow in B) to an almost complete loss of the posterior wing margin (black line in C). (D) A dpp-Gal4>Uif* adult wing shows wing margin loss (arrow) at the most distal tip area of the wing and an occasional loss of the anterior cross vein (arrowhead). (E and F) Thickened veins, which resemble an aspect of the Notch loss of function phenotypes (particularly veins III and V, arrows), observed in adult wings of *A9-Gal4*>Uif* (E) and MS1096-Gal4>Uif* (F) flies. (G–I) Expression of Uif* in the notal region causes losses of sensory bristles. (G) A wt adult notum with a regular pattern of sensory bristles. (H) The notum of Eq-Gal4>Uif* flies shows random losses of microchaeta (asterisks). (I) Expression of Uif* in the notum controlled by *pnr-Gal4* leads to a great loss of sensory bristles (rectangle).

To investigate the role of *uif* during development, we generated *UAS-Uif* transgenic flies that express an altered form of Uif, referred to as Uif*, which is a nearly full-length protein but has an altered intracellular domain (see Materials and Methods and below for details). We assumed initially that Uif* may act in a dominant negative manner, but further studies made possible by newly available tools revealed that its biological effects mirror those of the wild type (wt) Uif protein (see below). Ubiquitous expression of Uif* caused a semi-lethal phenotype (data not shown). To circumvent this lethality problem and facilitate the investigation of the role of *uif* in development, we used drivers to express Uif* in a tissue-specific manner. Ectopic expression of Uif* in the posterior compartment of the wing by *engrailed-Gal4* (*en-Gal4*) (*en-Gal4*>Uif*) resulted in significant tissue loss (Figure 1B and 1C). Defects were also observed when Uif* was expressed in other compartments of the wing. For example, *decapentaplegic-Gal4* (*dpp-Gal4*) driven expression of Uif* at the anterior-posterior (AP) boundary of the wing disc caused notched wing in the distal region of the wing margin (Figure 1D). In addition, expression of Uif* driven by *A9-Gal4* and *MS1096-Gal4* in the dorsal compartment of the wing led to thickened veins (Figure 1E and 1F). These results show that ectopic expression of Uif* causes patterning defects during development.

The wing phenotypes caused by the ectopic expression of Uif* are reminiscent of those caused by mutations affecting components of the Notch signaling pathway, suggesting that ectopically expressed Uif* may regulate Notch signaling. To evaluate this possibility, we further targeted Uif* expression in sensory organ precursor (SOP) cells. Notch signaling is required for SOP selection and formation [13], [14]. Consistent with the wing defects, Uif* expressed in SOP cells caused SOP selection and formation defects (Figure 1H and 1I), including patches of bristle loss (asterisks in Figure 1H, driven by *Eq-Gal4*) and a nearly complete loss of bristles in the notum and scutullem (rectangle in Figure 1I, driven by *pannier-Gal4* (*pnr-Gal4*)). Independent UAS-Uif* transgenic lines exhibited similar phenotypes (see Materials and Methods for details). These results show that ectopic expression of Uif* in two distinct tissues causes phenotypes that are characteristic of Notch signaling defects. Since the Uif*-induced defects in wing patterning and SOP selection were not mitigated by reducing a wt copy of *uif* (in *uif^6^*/+ heterozygotes; data not shown), we suggest that ectopically expressed Uif* acts in a neomorphic manner.

### Uif* genetically interacts with Notch pathway components

**Figure 2:**
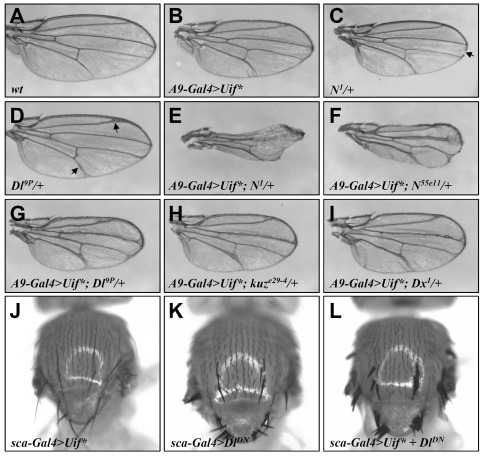
Uif* genetically interacts with genes for the Notch signaling pathway. (A) A wt adult wing. (B) An adult wing of the A9-Gal4>Uif* fly showing mild thickened vein phenotype that resembles *Dl* loss of function phenotype (D). (C) A *N^1^/+* wing showing a typical small notch at the distal region of the wing margin (arrow). (D) A *Dl^9P^/+* wing showing the thickened vein phenotype, particularly in the distal region of veins II and V (arrows). Wings of either *N^55e11^/+*, *kuz^e29-4^/+*, or *Dx^1^/+* adult flies have no or mild defects (Figure S1). (E–I) Wings of A9-Gal4>Uif* in combination with one copy of mutation of different Notch pathway components showing enhanced phenotypes as compared with either of them alone. Very small wings with a great loss of wing margin structures and thickened veins are shown in A9-Gal4>UAS-Uif*; N^1^/+ (E) and A9-Gal4>UAS-Uif*; N^55e11^/+ (F); blistering wing phenotype is also observed in the majority of adult flies (see Discussion). Thickened veins are shown in A9-Gal4>UAS-Uif*; Dl^9P^/+ (G) A9-Gal4>UAS-Uif*; kuz^e29-4^/+ (H) and A9-Gal4>UAS-Uif*; Dx^1^/+ (I) as compared with A9-Gal4>UAS-Uif* (B). (J–L) The notal region of an adult fly expresses UAS-Uif* (J), *UAS-Dl^DN^* (K) or *UAS-Dl^DN^* plus UAS-Uif* (L) under the control of *sca-Gal4*. The neurogenic phenotype of extra bristles caused by the loss of *Dl* function is potentiated by the simultaneous expression of Uif*.

To further investigate the role of Uif* in modulating the Notch pathway activity, we performed genetic interaction studies between Uif* and genes encoding Notch signaling components (Figure 2). A9-Gal4>Uif* adult flies had a weak thickened vein phenotype (Figure 2B; compare with *wt* Figure 2A) and *N^1^/+* wings had small notches at the wing margin (arrow in Figure 2C). However, the combination of *N^1^/+* and A9-Gal4>Uif* led to a much stronger phenotype, with a severe loss of wing margin structures and more thickened veins (Figure 2E). Another *Notch* allele, *N^55e11^*, which on its own only had a very mild wing defect as heterozygotes (Figure S1A), similarly exhibited genetic interaction with A9-Gal4>Uif*, leading to enhanced wing phenotypes (Figure 2F). The thickened vein phenotype of the *Dl^9P^/+* flies (typically for veins III and V, indicated by arrows in Figure 2D) was also synergistically enhanced by A9-Gal4>Uif*, with all veins becoming more broadened and the entire wing becoming smaller (Figure 2G).

In addition to Notch and Dl, we also analyzed two other components of the Notch pathway in genetic interaction experiments. Kuzbanian (Kuz) is a member of the ADAM family of metalloproteases and mediates S2 cleavage of Notch [15]. Deltex (Dx) is an E3-ubiquitin ligase, which binds to the intracellular domain of Notch and positively regulates Notch signaling [16]. While flies that are heterozygous for *Kuz* or *Dx* had no or mild wing phenotypes on their own (Figure S1B and S1C), introduction of A9-Gal4>Uif* into these flies led to significantly enhanced phenotype of thickened veins (Figure 2H and 2I; compare with Figure 2B for A9-Gal4>Uif* alone). Uif* also interacted genetically with genes for Notch pathway components in SOP development. In particular, the neurogenic phenotype of extra bristles caused by loss of *Dl* function was potentiated by a simultaneous expression of Uif* under the control of *sca-Gal4* (Figure 2J–2L). Together, these results document a genetic interaction between Uif* and genes encoding components of the Notch signaling pathway.

### Rescue of Uif*-induced defects by downstream components of the Notch signaling pathway

**Figure 3:**
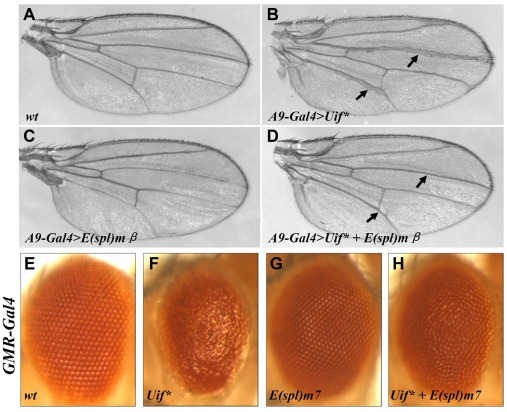
Expression of Notch target genes rescues Uif*-induced defects. (A) A wt wing. Expression of Uif* under the control of *A9-Gal4* causes thickened vein phenotype with broadened veins III and V (arrows in B). This defect can be significantly alleviated by coexpression of a Notch downstream component, E(spl)mβ (arrows in D). (C) shows control wing of *A9-Gal4*>E(spl)mβ flies. A small and rough eye phenotype (F) in *GMR-Gal4*>Uif* flies is significantly rescued by coexpression of E(spl)m7 (H). (E) and (G) show control eyes of *GMR-Gal4/+* and *GMR-Gal4>E(spl)m7* flies, respectively.

Previous studies have identified Notch downstream target genes that can specifically and selectively suppress phenotypic defects caused by mutations affecting Notch signaling in different tissues [17], [18]. If Uif* indeed exerts its biological effects by negatively impacting the Notch signaling pathway, coexpression of the relevant downstream components of the Notch pathway may rescue Uif*-induced defects. We tested this idea in both the wing and the eye. Our results show that the thickened vein phenotype of *A9-Gal4*>Uif* adult wings (arrows in Figure 3B) was almost completely suppressed by *A9-Gal4*>*E(spl)mβ* (Figure 3D), which on its own caused slightly thinner veins (Figure 3C and [19]). Furthermore, the rough and small eye phenotype of the GMR-Gal4>Uif* flies (Figure 3F) was significantly alleviated by coexpression of E(spl)m7 (Figure 3H), which on its own did not have any detectable abnormality (Figure 3G). These results, together with those shown in Figure 2, further support the hypothesis that Uif* perturbs developmental processes through its inhibitory effects on the canonical Notch signaling pathway.

### Expression of Uif* affects the expression of Notch target genes

**Figure 4:**
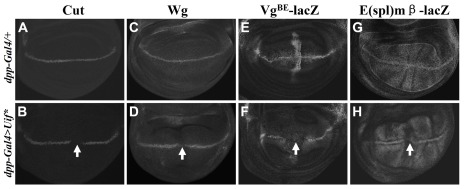
Uif* reduces the expression of Notch target genes. Expression of Notch target genes, Cut (A and B), Wg (C and D), vg^BE^-lacZ (E and F) and E(spl)mβ–lacZ (G and H), in the third instar wing discs of wild type larvae, with (B, D, F and H) or without (A, C, E and G) Uif* overexpression. Genotypes are: (A and C) *dpp-Gal4 UAS-GFP/+*; (B and D) dpp-Gal4 UAS-GFP/UAS-Uif*; (E) *dpp-Gal4 UAS-GFP/vg^BE^-lacZ*; (F) dpp-Gal4 UAS-GFP/vg^BE^-lacZ UAS-Uif*; (G) *E(spl)mβ–lacZ/+; dpp-Gal4 UAS-GFP/+* and (H) E(spl)mβ–lacZ/+; dpp-Gal4 UAS-GFP/UAS-Uif*. Arrows indicate a loss or a decreased expression of the Notch target genes at the AP boundary of the wing discs where Uif* was expressed under the control of *dpp-Gal4* (B, D, F and H).

Notch signaling controls wing margin formation by activating its downstream target genes, such as *cut*, *wingless* (*wg*) and *vestigial* (*vg*), in a stripe of cells along the dorsal-ventral (DV) boundary of the third instar larvae wing imaginal discs [20], [21], [22], [23]. To investigate at a molecular level the effect of Uif* on Notch signaling, we analyzed the expression patterns of Notch target genes. Two of these target genes (Figure 4A and 4C), *wg* and *cut*, are known to respond to low and high thresholds of Notch signaling activity, respectively [11]. Our results show that, consistently, while Wg expression was significantly reduced by *dpp-Gal4* directed ectopic expression of Uif* at the AP boundary (Figure 4D, arrow), Cut expression was completely eliminated (Figure 4B, arrow). In addition to endogenous target genes of Notch, we also analyzed two reporter genes that contain Su(H) binding sites, *vg^BE^-lacZ* and *E(spl)mβ-lacZ* [22], [24]. Figure 4F and 4H show that the expression of both reporters was also significantly decreased at the AP boundary where *dpp-Gal4* expresses (arrows). Together, these results provide molecular evidence that expression of Uif* directly affects the activity of the canonical Notch signaling pathway.

### Full-length Uif can similarly antagonize Notch signaling

**Figure 5:**
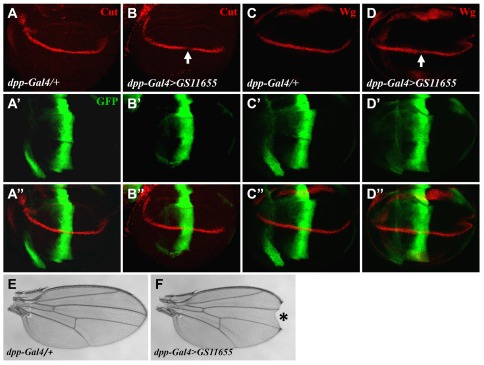
Full-length wt Uif antagonizes Notch signaling. Overexpression of wt Uif by *dpp-Gal4*>*GS11655* leads to a significant reduction in the levels of both Cut and, to a lesser degree, Wg. (A) and (C) show Cut and Wg expression patterns in wing discs from the *dpp-Gal4/+* control flies, respectively. (B) and (D) show the Cut and Wg levels in wing discs from *dpp-Gal4*>*GS11655* flies, respectively (see regions pointed by arrows). GFP in (A′–D′) shows the expression pattern of *dpp-Gal4*. A notched wing detected in a *dpp-Gal4*>*GS11655* adult fly (F), compared with a *dpp-Gal4/+* control wing (E). Flies were reared at 29°C.

Uif* is almost a full-length form of the protein, with its C-terminal ten amino acids truncated (see Materials and Methods for details). It is well documented that removing the intracellular domains of Dl and Ser can generate dominant negative forms of these ligands [25]. To determine whether the defects caused by Uif* might be due to a similar dominant negative effect, we employed a recently available transgenic fly (*GS11655* from the Kyoto *Drosophila* Genetic Resource Center) that harbors Gal4-binding sites upstream of the endogenous wt *uif* gene. Antibody staining shows that this endogenous *uif* gene can respond to the *dpp-Gal4* driver leading to an increased wt Uif level (Figure S2A). In wing discs of *dpp-Gal4>GS11655* flies, Cut protein level was significantly reduced at the AP boundary (arrow in Figure 5B). The inhibitory effect of wt Uif on Wg expression was also detectable (arrow in Figure 5D) but, as expected, weaker than that on Cut. In addition, we detected notched wings in adults expressing wt Uif under the control of *dpp-Gal4* (asterisk in Figure 5F). Together, these results show that overexpression of wt Uif causes molecular and phenotypic defects that are similar to those of Uif*. However, the effects of Uif* are stronger than wt Uif (Figure S3), which we attribute to the higher accumulated levels of Uif* in our experiments (Figure S2 and Discussion). An important finding here is that these results argue against the possibility that Uif* acts merely, if at all, as a dominant negative form of the protein due to its altered intracellular domain, suggesting that ectopically expressed Uif* and wt Uif are functionally equivalent (though different in strengths) with respect to the regulation of Notch signaling.

### Uif* inhibits Notch signaling at a step dependent on the extracellular domain of Notch

**Figure 6:**
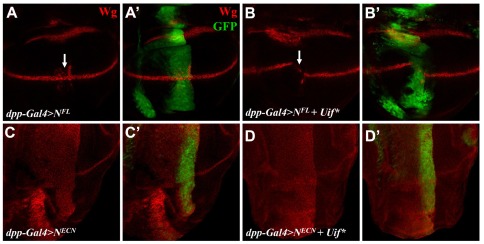
The inhibitory effect of Uif* is dependent on the extracellular domain of Notch. Ectopic expression of the full-length Notch (N^FL^) under the control of *dpp-Gal4* induces aberrant Wg (red) expression at the AP boundary where it intersects with the DV boundary (white arrow in A) (A and A′). GFP (green) marks *dpp-Gal4* positive cells (A′, B′, C′ and D′). Coexpression of Uif* with N^FL^ reduces the ectopic induction of Notch signaling mediated by N^FL^ at the intersection between AP and DV boundaries (white arrow in B) (B and B′). Ectopic expression of the membrane tethered active version of Notch (N^ECN^) induces Wg (red) expression in the *dpp-Gal4* region that is marked by GFP (green) (C and C′). Coexpression of Uif* does not alter the Wg expression that is induced by N^ECN^ (D and D′).

Similar to the *Drosophila* Notch ligands, Dl, Ser and Wry, Uif also contains EGF-like repeats. It has been shown that the EGF-like repeats of the Notch ligands directly interact with the extracellular domain of Notch [26]. To determine whether Uif may antagonize Notch signaling in a manner that is dependent on the extracellular domain of Notch, we compared the effects of Uif* on Notch receptors that either have or lack this domain. Expression of full-length Notch (N^FL^) (driven by *dpp-Gal4*) ectopically activated Notch target genes at the AP boundary close to the DV boundary (arrow in Figure 6A; [21]). As expected, this ectopic target gene expression was significantly suppressed by coexpression of Uif* (Figure 6B). However, Uif* had no effect on a constitutively active form of Notch that lacks its extracellular domain (N^ECN^) and activated its downstream target genes in a ligand-independent manner (compare Figure 6D with 6C). These results suggest that Uif modulates Notch signaling at a step that is dependent on the extracellular domain of Notch.

### Uif* inhibits Notch signaling through a *cis* mechanism

**Figure 7:**
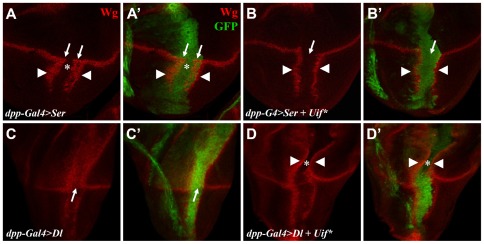
Uif* enhances *cis* inhibition of Notch signaling by its ligands. (A and A′) *dpp-Gal4>UAS-Ser* leads to both *cis* inhibition (in the inner region of the *dpp-Gal4* expressing, GFP^+^ domain in the ventral part of the disc; marked by the asterisk) and *trans* activation of Wg (in cells neighboring to the *dpp-Gal4* expressing domain in the ventral compartment of wing disc; marked by arrowheads). The *cis* inhibition is incomplete and, thus, Wg expression (arrows) is detected in the outer region of the *dpp-Gal4* expressing domain. Coexpession of Uif* enhances *cis* inhibition, leading to Wg reduction inside the *dpp-Gal4* expressing domain, without affecting *trans* activation (arrowheads in B and B′). Expression of Dl by *dpp-Gal4* causes Wg expression mainly in the dorsal compartment both inside and outside of the *dpp-Gal4* regions (C). Wg protein level inside of the *dpp-Gal4* expressing domain is lower, reflective of *cis* inhibition (arrow in C). When Uif* is coexpressed, this *cis* inhibition is enhanced, leading to a nearly complete loss of Wg expression inside of the *dpp-Gal4* expression domains (asterisks in D and D′), without affecting *trans* activation (outside of *dpp-Gal4* expression domain; arrowheads in D and D′). GFP (green) marks the domain where *dpp-Gal4* is expressed (A′, B′, C′ and D′). See the main text for further details.

The ligands Dl and Ser can regulate Notch signaling through both paracrine and autocrine interactions [20], [21], [27], [28], [29], [30], [31], [32], [33]. Paracrine interaction leads to Notch activation *in trans* (referred to as *trans* activation) whereas autocrine interaction leads to Notch inhibition *in cis* (referred to as *cis* inhibition). Both effects are achieved through physical interactions between the extracellular domains of Notch and its ligands [31]. To further clarify the nature of Uif* action with regard to its topological relationship with Notch, we took advantage of an ectopic expression system, where both *cis* inhibitory and *trans* activating effects of Notch ligands are exhibited simultaneously [34], [35]. Here, we used *dpp-Gal4* to drive Ser or Dl ectopic expression in the wing imaginal discs (Figure 7). In addition to *trans* activation exhibited by the ectopic Wg expression, the *cis* inhibitory effects of these ligands were simultaneously exhibited by a reduction of Wg expression levels within the domain of ligand-expressing cells (marked by GFP; asterisk in Figure 7A and arrow in Figure 7C). However, such *cis* inhibitory effects are incomplete and detectable only in cells expressing ligands at high levels. Coexpression of Uif* with the ligands greatly enhanced the *cis* inhibition, leading to a complete elimination of Wg expression in almost all ligand-expressing cells (arrow in Figure 7B and asterisk in 7D). These results suggest that ectopic expression of Uif* negatively regulates Notch signaling through a *cis* inhibitory mechanism, either working on its own or, more likely (as in our experimental setting), working in concert with Ser or Dl.

## Discussion

The canonical Notch signaling pathway is one of a limited group of pathway modules that transduce signals from outside the cell to alter gene expression inside the nucleus [1], [2], [3]. These pathways together orchestrate the developmental processes that can be dauntingly complex. Yet it is the same modules that are used repeatedly, not only in different organisms, but also in vastly different processes within an organism [4], [36]. Thus, how these pathway modules are activated in a specific manner, with regard to not only space and time but also the quantity of their signaling output, represents a fundamental question in developmental biology. Here we describe a newly characterized protein, Uif, which can antagonize the canonical Notch signaling pathway in a neomorphic manner. These findings underscore the importance of the precise tuning of Notch activity in normal patterning.

EGF-like repeats are a common feature of Notch receptors, ligands and co-ligands [6], [37]. While Uif was originally characterized for its role in tracheal development, its EGF-like repeats suggest a possible role in Notch signaling. Our results are consistent with a model where ectopically expressed Uif may modulate the accessibility of the extracellular domain of Notch to its ligands during activation. It is possible that the EGF-like repeats of Uif directly interact with the extracellular domain of Notch to exert its inhibitory effect in a manner similar to the *cis* inhibition by Notch ligands themselves [20], [28], [29], [30], [31], [32], [33]. Our finding that Uif* acts on Notch through a *cis* inhibitory mechanism (Figure 7) is supportive of this possibility. In our experiments, Uif* is more effective than wt Uif in antagonizing Notch, and this difference may be attributed to the difference in their expression levels (Figure S2). These results suggest that ectopically expressed Uif* and wt Uif have a similar neomorphic function in regulating Notch signaling.

A proposed neomorphic function of Uif* and Uif in Notch signaling is consistent with our results of loss of function analysis of *uif*. Knockdown (assayed for adult wing phenotypes and Notch target gene expression using independent RNAi lines; data not shown) or knockout (assayed for Notch target gene expression in somatic mutant clones; Figure S4) of *uif* revealed neither Notch loss of function nor gain of function phenotypes. However, it remains formally possible that the endogenous *uif* gene has a native role in regulating Notch signaling in tissues or cells (other than those that we have examined) at a time during *Drosophila* development. Further studies are required to investigate this possibility.

The biological activities of Uif are not restricted to regulating Notch signaling. The fact that Uif was originally characterized for its role in tracheal inflation underscores the complexity of its biological activities. In addition to the EGF-like repeats, Uif also contains several other domains that may have important biological functions. These domains include a C-type lectin-like (CLECT) domain, three CUB domains, eight complement control protein (CCP) domains, two coagulation factor 5/8 C-terminal (FA58C) domains and three hyaline repeat (HYR) domains. Both CLECT and FA58C domains are putative carbohydrate binding domains known to play important roles in many diverse processes [38], [39]. The CUB domain is an evolutionary conserved protein domain found almost exclusively in extracellular and plasma membrane-associated proteins [40]. HYR is an immunoglobulin fold domain likely involved in cell adhesion [41]. The CCP domains, also known as the Sushi domains or Short Consensus Repeats (SCR), exist in a wide variety of complement and adhesion proteins [42]. These domains suggest that Uif may also play a role in cell adhesion. Indeed, in a recent genetic modifier screen, *uif* was identified as a regulator (*Mod29*) of the *Drosophila* Dystroglycan-Dystrophin Complex, a specialized cell adhesion complex [43]. Mod29/Uif was suggested to play roles in multiple developmental processes, including wing vein formation, muscle and photoreceptor axon development, and oogenesis [43]. Although it remains to be investigated whether Uif, a large regulator with multiple conserved protein domains, may functionally connect distinct cellular processes, our own unpublished data offer some speculative insights. In particular, the blistering wing phenotype caused by knockdown of *Dl* or *Ser* [44] can be fully rescued by depletion of *uif* (data not shown), suggesting that Uif may functionally extend the role of Notch ligands to cell adhesion. Uif is an N-glycosylated protein, a modification shared by several proteins known to play a role in the formation of large protein complexes [45]. Understanding the full spectrum of the biological functions of Uif during development and, importantly, its potential role in harmonizing different cellular processes, represents future challenges.

## Materials and Methods

### Generation of UAS-Uif* and *UAS-Uif^RNAi^* transgenic flies

A pUAST-Uif* construct was made by inserting a part of the *uif* cDNA sequence that encodes the first 165 amino acids of Uif and a genomic DNA fragment encoding the remaining amino acids of Uif-PA into the pUAST vector. This transgene is expected to encode a protein that lacks the last ten amino acids at the C-terminus of the predicted full-length Uif protein (amino acid 3548 to amino acid 3557), with two amino acid changes (N1567D and A3134T) and an addition of three extra amino acids (SGR) immediately after amino acid 165 resulting from the insertion of a restriction enzyme Not I site in the coding sequence. After standard P element-mediated germline transformation, three independent lines of transgenic flies that carry pUAST-Uif* were obtained, all of which resulted in similar phenotypes when expressed under different Gal4 drivers tested. Immunostaining with antibodies against extracellular and intracellular domains of Uif demonstrated that Uif* is properly and stably expressed under the control of *dpp-Gal4* (Figure S2B and data not shown).

To construct *UAS-Uif^RNAi^* flies, two pieces of non-overlapping *uif* coding sequence were cloned into the pWIZ vector [46]. Germline transformants that carry each sequence were generated by standard procedures at the Rainbow Transgenic Flies Inc (Camarillo, CA). At least three independent lines for each RNAi constructs were tested for the RNAi strength and specificity.

### Null mutant of *uif* and other *Drosophila* strains

*uif* null mutants were generated by homologous recombination mediated gene targeting strategy [47], [48]. One of the alleles, designated *uif^6^*, which was molecularly verified and can be completely rescued by a genomic transgene of *uif* (Figure S4 and data not shown), was used in this study. The transgenic fly strain used for genomic rescue was generated by direct injection of a BAC clone (CH321-83F13, from P[acman] BAC libraries, BPRC (BACPAC Resources Center)) that contains the *uif* genomic fragment into flies, which harbor both the *vas-phiC31* transgene (on X chromosome) and a *attP* target site (on 3^rd^ chromosome) [49]. Other fly strains that were used in this study include: *w^1118^*, UAS-Uif* (this paper), *Eq-Gal4* [50], *pnr-Gal4*, *Dl^9P^/TM3 Sb*, *N^1^*, *N^55e11^*, *kuz^e29-4^*, *Dx^1^*, *sca-Gal4*, *UAS-Dl^DN^*, *y^1^ w^67c23^; P{w[+mC] = GSV6}GS11655/SM1* (DGRC#203493, Kyoto), *en-Gal4*, *GMR-Gal4*, *UAS-GFP*, *dpp-Gal4*, *MS1096-Gal4*, *A9-Gal4*, *vg^BE^-lacZ* [34], *E(spl)mβ-lacZ*, *y w hsFlp^122^*; *ubi-GFP FRT40A/CyO*, *UAS-Uif^RNAi-1^*, *UAS-Uif^RNAi-2^*, *UAS-E(spl)mβ*, *UAS-E(spl)m7*, *UAS-N^FL^*, *UAS-N^ECN^* [51], *UAS-Dl^30^* [51] and *UAS-Ser*. All flies were from the Bloomington *Drosophila* Stock Center at Indiana University unless otherwise stated. All crosses were carried out at 25°C according to standard procedures unless stated otherwise.

### Generation of anti-Uif antibodies

We generated antibodies against the extracellular domain and the intracellular domain of Uif. Briefly, *uif* coding sequences for amino acids 1113–1343 (extracellular domain) and 3440–3548 (intracellular domain) were cloned into the pET21b(+) vector. The proteins were expressed in BL21 *E. coli* cells and purified according to Qiagen Ni-NTA handbook. Purified proteins were used to generate antibodies in rabbits at the Cocalico Biologicals Inc (Reamstown, PA). The anti-Uif sera were subsequently affinity purified with protein G beads (Invitrogen) prior to use in immunostaining (1∶500).

### Immunohistochemistry

Immunostaining of wing imaginal discs was performed as previously described [52], [53]. In addition to antibodies against Uif (see above), the following primary antibodies were used: mouse anti-Wg (4D4, 1∶20, the Developmental Studies Hybridoma Bank [DSHB], University of Iowa, Iowa City, IA, USA), mouse anti-Cut (2B10, 1∶20, DSHB), rabbit anti-GFP (1∶1000, Invitrogen) and rabbit anti-β-Galactosidase (1∶1000; Sigma). The secondary antibodies used were conjugated to FITC or Cy3 (Jackson Immunoresearch), each diluted at 1∶200. Images were captured on a Leica TSC SP5 confocal laser scanning microscope and processed using Adobe Photoshop.

## Supporting Information

Figure S1 **Adult wings of** ***N^55e11^*** **/+,** ***kuz^e29-4^/+*** **and** ***Dx^1^/+*** **heterozygous flies.** (A) An adult wing of *N^55e11^*/+ flies shows a mild delta vein phenotype in the most distal regions of veins IV and V (arrows; compare with a wt wing in Figure 1A). (B and C) *kuz^e29-4^/+* and *Dx^1^/+* adult wings have normal wing pattern.
(TIF)

Figure S2 Wild type Uif and Uif* are expressed at different levels in the wing disc. Wing discs immunnostained with anti-Uif antibody showing the ectopic expressing level of wt Uif (A and A″) or Uif* (B and B″). GFP marks the *dpp-Gal4* expressing cells in A′, A″, B′ and B″. All experiments shown here were performed side by side with images captured and processed under identical settings. Flies were reared at 18°C.
(TIF)

Figure S3 Comparison of the effects of wt Uif and Uif* on Cut expression. (A) Cut expression at the DV boundary in the control wing disc (*dpp-Gal4*/+). Expression of wt Uif by *dpp-Gal4>GS11655* causes a detectable reduction of the Cut level at the AP boundary (arrow in B). Panel C shows a stronger reduction of Cut expression caused by Uif* (arrow). GFP marks *dpp-Gal4* expressing cells. All experiments shown here were performed side by side with images captured and processed under identical settings.
(TIF)

Figure S4 **Notch signaling is not detectably upregulated in** ***uif*** **mutant clones.** (A) Cut expression pattern in the wing disc with FRT40A mock clones, marked by the absence of GFP (A′). (B) No detectable changes of Cut expression pattern in the *uif^6^* mutant clones (marked by GFP negative cells in B′) comparing with the mock clones. (A″ and B″) are the overlaid images. (D) Adult wing with *uif^6^* mutant clones show wrinkles and reduced size as compared with wild type (C), which is fully rescued by a copy of *uif* genomic DNA (E).
(TIF)
