# Supplementary figures and images for: Uif, a Large Transmembrane Protein with EGF-Like Repeats, Can Antagonize Notch Signaling in Drosophila
Source: PLoS One. 2012 Apr 30;7(4):e36362. doi: 10.1371/journal.pone.0036362 (PMC3340373; doi:10.1371/journal.pone.0036362)

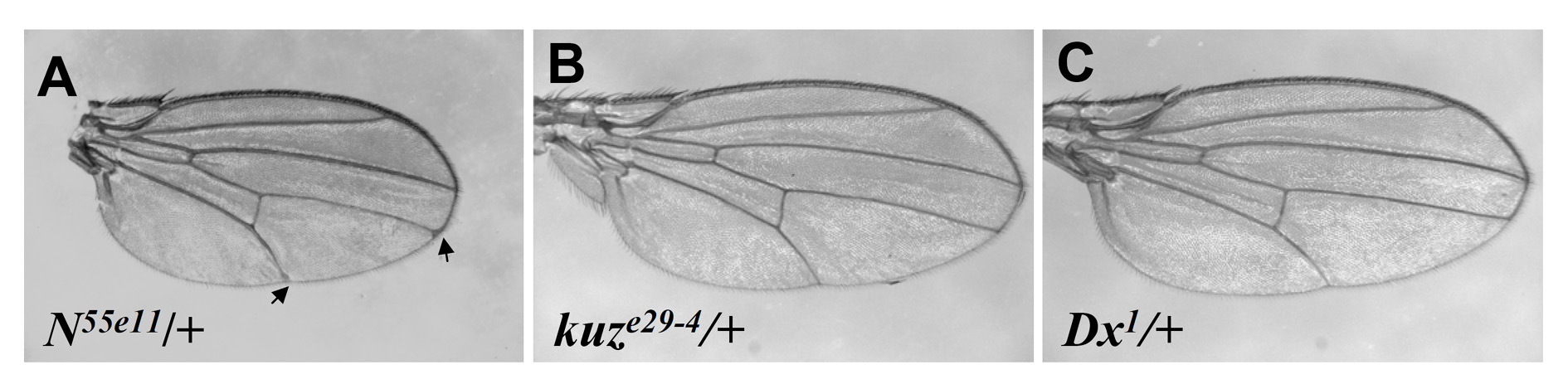

Supplement: Figure S1 — Adult wings of N55e11 /+, kuze29-4/+ and Dx1/+ heterozygous flies. (A) An adult wing of N55e11/+ flies shows a mild delta vein phenotype in the most distal regions of veins IV and V (arrows; compare with a wt wing in Figure 1A). (B and C) kuze29-4/+ and Dx1/+ adult wings have normal wing pattern. (TIF) [file pone.0036362.s001.tif]

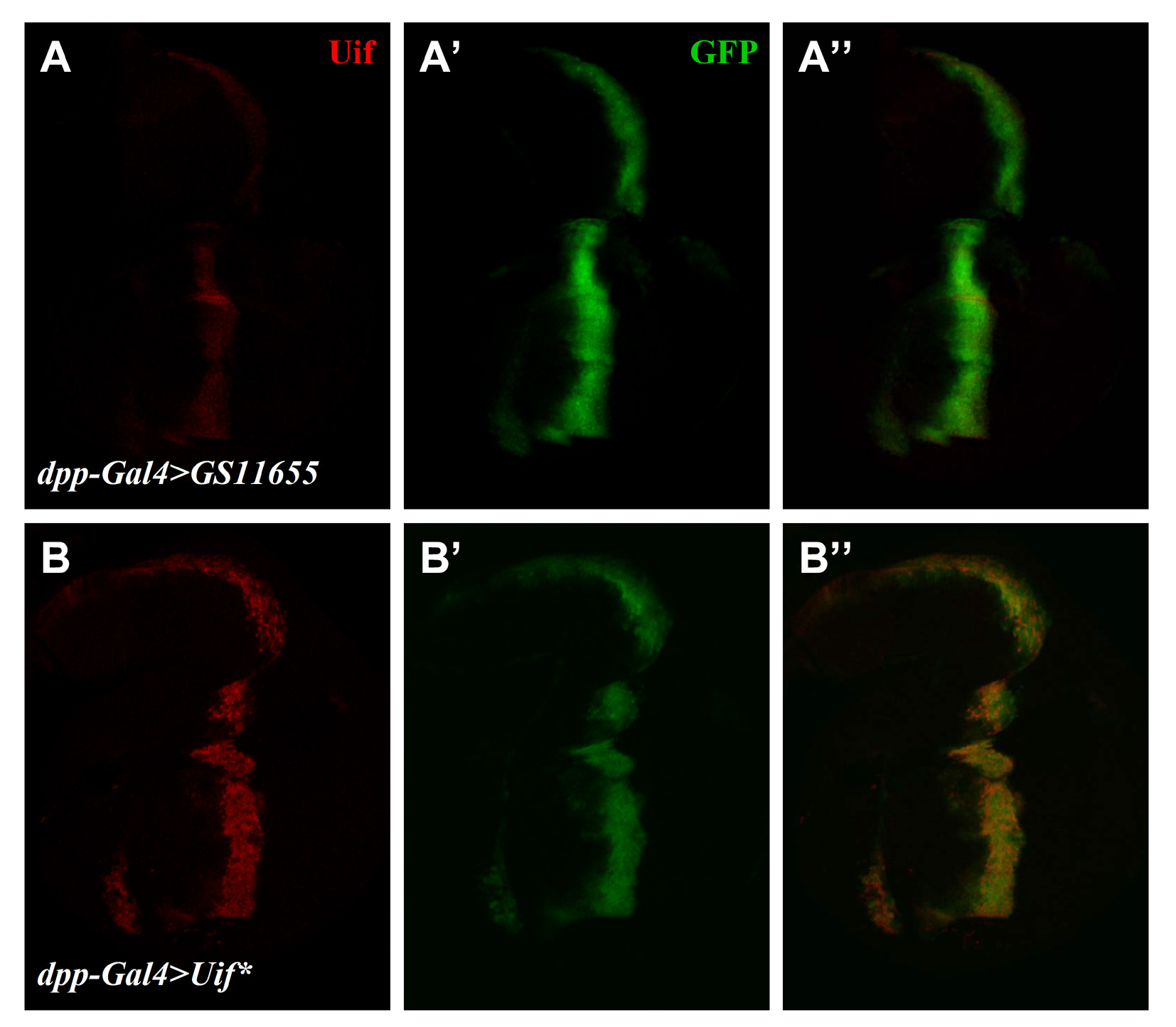

Supplement: Figure S2 — Wild type Uif and Uif* are expressed at different levels in the wing disc. Wing discs immunnostained with anti-Uif antibody showing the ectopic expressing level of wt Uif (A and A″) or Uif* (B and B″). GFP marks the dpp-Gal4 expressing cells in A′, A″, B′ and B″. All experiments shown here were performed side by side with images captured and processed under identical settings. Flies were reared at 18°C. (TIF) [file pone.0036362.s002.tif]

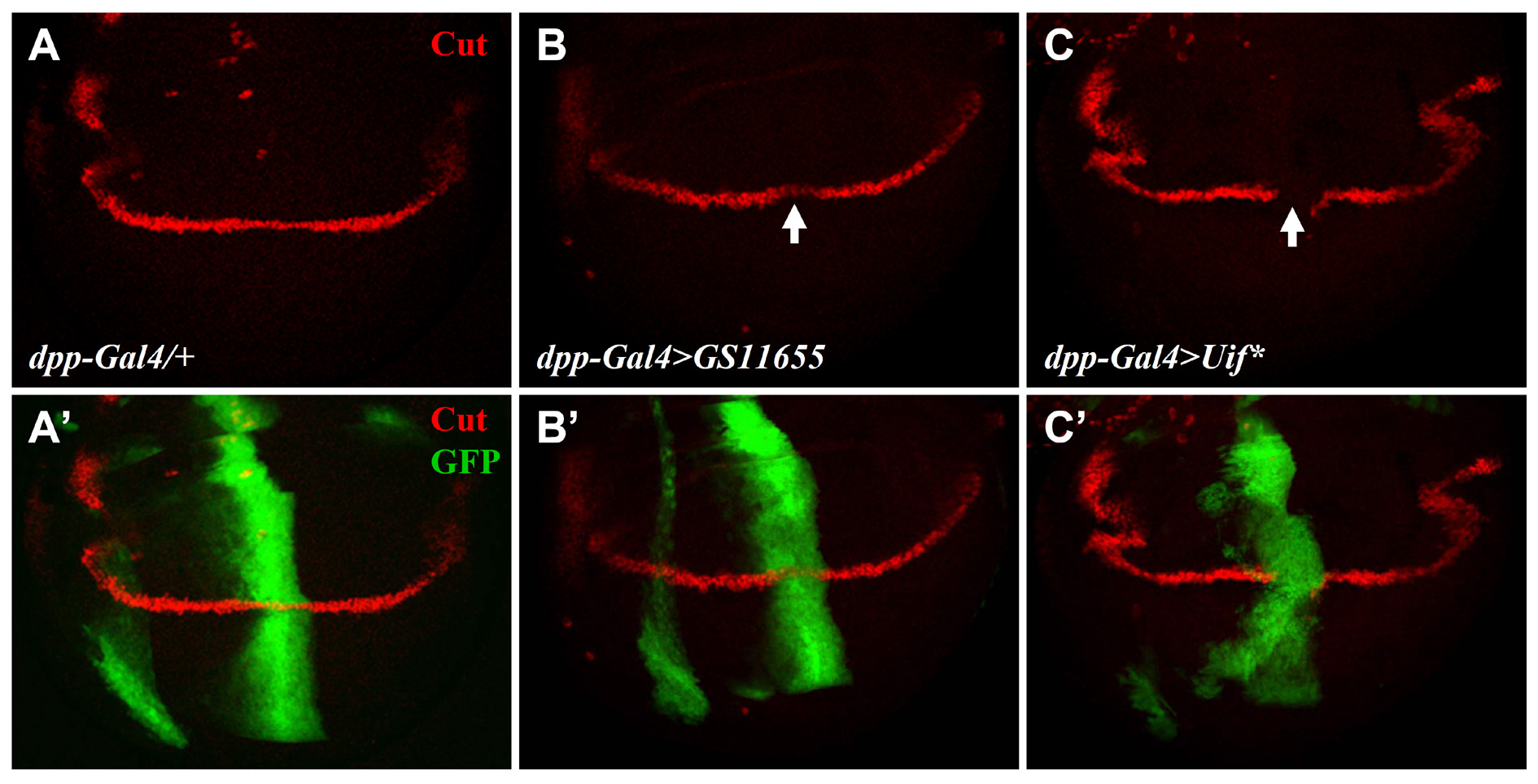

Supplement: Figure S3 — Comparison of the effects of wt Uif and Uif* on Cut expression. (A) Cut expression at the DV boundary in the control wing disc (dpp-Gal4/+). Expression of wt Uif by dpp-Gal4>GS11655 causes a detectable reduction of the Cut level at the AP boundary (arrow in B). Panel C shows a stronger reduction of Cut expression caused by Uif* (arrow). GFP marks dpp-Gal4 expressing cells. All experiments shown here were performed side by side with images captured and processed under identical settings. (TIF) [file pone.0036362.s003.tif]

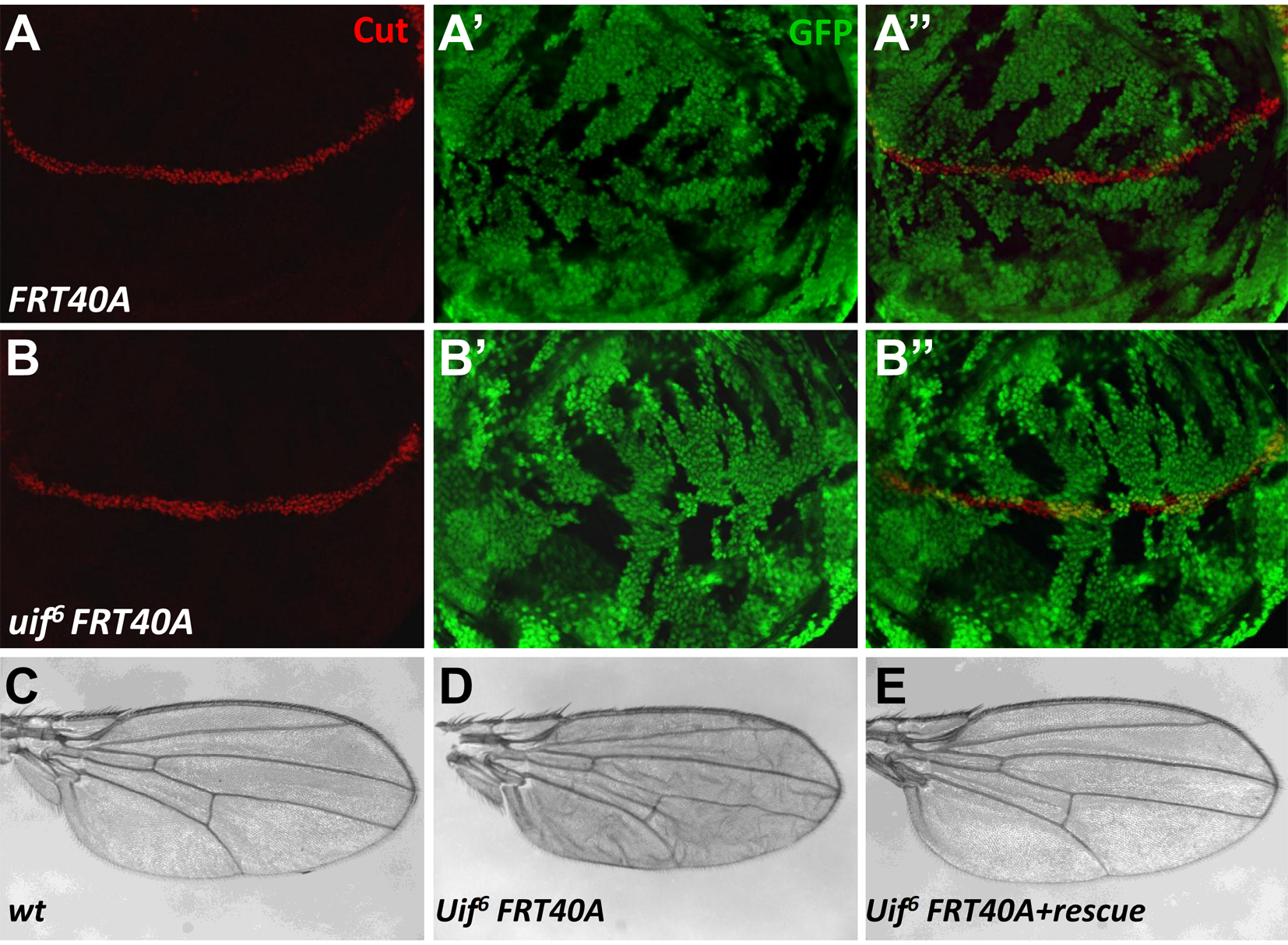

Supplement: Figure S4 — Notch signaling is not detectably upregulated in uif mutant clones. (A) Cut expression pattern in the wing disc with FRT40A mock clones, marked by the absence of GFP (A′). (B) No detectable changes of Cut expression pattern in the uif6 mutant clones (marked by GFP negative cells in B′) comparing with the mock clones. (A″ and B″) are the overlaid images. (D) Adult wing with uif6 mutant clones show wrinkles and reduced size as compared with wild type (C), which is fully rescued by a copy of uif genomic DNA (E). (TIF) [file pone.0036362.s004.tif]
